# Supplementary material for: Comparative Study on Production Performance of Different Oat (Avena sativa) Varieties and Soil Physicochemical Properties in Qaidam Basin
Source: Plants (Basel). 2025 Jun 28;14(13):1978. doi: 10.3390/plants14131978 (PMC12252256; doi:10.3390/plants14131978)
Supplement: Supplementary file 1 [file plants-14-01978-s001.zip › plants-3700390-supplementary.pdf]

Table S1. Growth stage of different oat varieties

Variety	Phenological period (month-day)		
	**Sowing stage**	**Seedling stage**	**Harvest period**
Qingtian No. 1	June 13 th	June 24 th	September 21 th
Molasses	June 13 th	June 22 th	September 23 th
Qinghai sweet oat	June 13 th	June 23 th	October 3 th
Qingyin No. 1	June 13 th	June 22 th	September 24 th
Qingyin No. 2	June 13 th	June 22 th	September 23 th
Dafuweng	June 13 th	June 23 th	October 5 th
Baiyan No. 7	June 13 th	June 24 th	September 20 th
Northwestern No. 1	June 13 th	June 26 th	September 12 th
Lena	June 13 th	June 23 th	September 15 th
Jiayan No. 2	June 13 th	June 23 th	September 22 th
Mengshi No. 1	June 13 th	June 21 th	September 19 th
Qinghai No. 444	June 13 th	June 22 th	September 9 th
Haymaker	June 13 th	June 22 th	September 10 th
Gaoyan No. 1	June 13 th	June 28 th	October 8 th
Baler	June 13 th	June 27 th	October 6 th

Table S2. Comparison of the effects of different oat varieties on soil organic matter

variety	Seedling stage 0-15cm organic matter /g·kg-1	Harvest period 0-15cm organic matter /g·kg-1	Seedling stage 15-30cm organic matter /g·kg-1	Harvest period 15-30cm organic matter /g·kg-1
CK	7.61±0.28hi	8.03±0.66i	4.08±0.61f	3.77±0.13f
Qingtian No. 1	12.07±0.55bc	14.1±0.33de	6.32±0.05bcd	9.88±1.08a
Molasses	10.57±0.17e	11.68±0.5g	6.14±0.34cd	8.08±1.23bc
Qinghai sweet oat	12.87±0.76ab	15.73±0.37b	6.22±0.46cd	8.64±0.96ab
Qingyin No. 1	13.2±0.57a	16.13±0.76b	6.28±0.39cd	8.34±0.89bc
Qingyin No. 2	10.87±0.42e	13.51±0.53ef	6.29±0.83cd	8.42±0.4abc
Dafuweng	9.55±0.15f	10.06±0.7h	6.45±0.17bcd	7.81±0.72bc
Baiyan No. 7	11.83±0.58cd	12.9±0.62f	7.26±0.52ab	8.06±1.04bc
Northwestern No. 1	8.26±0.38gh	17.23±0.41a	5.52±0.03de	7.36±0.64bcd
Lena	8.54±0.4g	11.45±0.31g	4.45±0.05f	5.17±0.06ef
Jiayan No. 2	7.48±0.34hi	14.13±0.54de	4.51±0.38f	5.23±0.84ef
Mengshi No. 1	12.22±0.62bc	15.3±0.28bc	6.47±0.36bcd	6.99±0.74cd
Qinghai No. 444	8.4±0.38gh	9.4±0.36h	5±0.7ef	5.9±0.49de
Haymaker	6.72±0.35i	10.1±0.29h	5.79±0.62cde	7.07±0.66cd
Gaoyan No. 1	10.37±0.29ef	14.64±0.46cd	7.7±0.29a	8.77±0.29ab
Baler	11.02±0.38de	15.7±0.08b	6.66±0.75bc	7.03±0.35cd
F Value	41.151**	60.204**	9.03**	9.04**

Note : CK is saline-alkali open space control. The values in the table are mean±standard error, different letters indicate significant differences among different breeds in the same column ($P<0.05$). ** indicates highly significant differences at the 0.01 level; * indicates significant differences at the 0.05 level.

Table S3. Comparison of the effects of different oat varieties on soil total nitrogen

variety	0-15cm total nitrogen /g·kg-1		15-30cm total nitrogen /g·kg-1	
	Seedling stage	Harvest period	Seedling stage	Harvest period
CK	0.49±0.02fg	0.53±0.03i	0.33±0.02i	0.37±0.01g
Qingtian No. 1	0.63±0.03ab	0.78±0.02a	0.37±0.01ij	0.59±0.04bc
Molasses	0.41±0.03i	0.67±0.03def	0.37±0.01hij	0.49±0.04ef
Qinghai sweet oat	0.69±0.01a	0.72±0.02bcd	0.50±0.01bc	0.57±0.00cd
Qingyin No. 1	0.52±0.02defg	0.77±0.04ab	0.34±0.01i	0.62±0.05bc
Qingyin No. 2	0.52±0.04efg	0.64±0.02efg	0.46±0.01cde	0.50±0.04e
Dafuweng	0.42±0.03hi	0.68±0.04cde	0.41±0.02fgh	0.42±0.03g
Baiyan No. 7	0.66±0.03a	0.78±0.04a	0.56±0.03a	0.64±0.04b
Northwestern No. 1	0.55±0.02cdef	0.77±0.03ab	0.39±0.00ghi	0.50±0.03de
Lena	0.46±0.04ghi	0.58±0.02hi	0.35±0.00ij	0.41±0.04g
Jiayan No. 2	0.48±0.03fgh	0.73±0.03abc	0.43±0.01efg	0.43±0.04fg
Mengshi No. 1	0.54±0.04cdef	0.65±0.04efg	0.47±0.04cd	0.50±0.03e
Qinghai No. 444	0.59±0.04bc	0.60±0.02gh	0.46±0.03de	0.50±0.03e
Haymaker	0.53±0.04cdef	0.61±0.03fgh	0.43±0.01efg	0.60±0.04bc
Gaoyan No. 1	0.59±0.03bcd	0.65±0.02efg	0.54±0.01ab	0.83±0.03a
Baler	0.57±0.02bcde	0.68±0.03cde	0.45±0.04def	0.51±0.03de
F Value	12.743**	14.118**	23.594**	24.795**

Note : CK is saline-alkali open space control. The values in the table are mean±standard error, different letters indicate significant differences among different breeds in the same column ($P<0.05$). ** indicates highly significant differences at the 0.01 level; * indicates significant differences at the 0.05 level.

Table S4. Comparison of effects of different oat varieties on soil Ca^{2+}

variety	0-15cm soil Ca^{2+}/（g·kg^{-1}）		15-30cm soil Ca^{2+}/（g·kg^{-1}）	
	Seedling stage	Harvest period	Seedling stage	Harvest period
CK	0.31±0.01a	0.26±0.02a	0.28±0a	0.24±0a
Qingtian No. 1	0.09±0.01f	0.04±0.01e	0.1±0.01g	0.05±0.01h
Molasses	0.09±0.01f	0.04±0.01e	0.07±0.01h	0.05±0h
Qinghai sweet oat	0.14±0.01de	0.09±0.01c	0.12±0.01fg	0.07±0.01ef
Qingyin No. 1	0.17±0c	0.08±0c	0.08±0h	0.06±0.01gh
Qingyin No. 2	0.14±0de	0.05±0e	0.11±0.01g	0.09±0d
Dafuweng	0.12±0.01e	0.08±0.01cd	0.18±0.01bc	0.09±0de
Baiyan No. 7	0.19±0.01b	0.12±0.01b	0.19±0.01b	0.12±0.01bc
Northwestern No. 1	0.12±0e	0.11±0.01b	0.14±0.01de	0.12±0.01c
Lena	0.1±0.01f	0.08±0.01c	0.11±0.01g	0.09±0.01de
Jiayan No. 2	0.16±0c	0.05±0e	0.16±0.01cd	0.13±0.01bc
Mengshi No. 1	0.12±0.01e	0.06±0de	0.11±0.01g	0.06±0.01fgh
Qinghai No. 444	0.14±0.01d	0.06±0.01de	0.07±0.01h	0.07±0.01fg
Haymaker	0.18±0b	0.07±0.01cd	0.1±0.01g	0.09±0de
Gaoyan No. 1	0.09±0f	0.09±0c	0.18±0bc	0.14±0b
Baler	0.1±0.01f	0.08±0.01c	0.13±0.01ef	0.09±0.01de
F Value	81.22**	54.61**	60.44**	71.77**

Note: CK is saline-alkali open space control. The values in the table are mean±standard error, different letters indicate significant differences among different breeds in the same column (P<0.05). ** indicates highly significant differences at the 0.01 level; * indicates significant differences at the 0.05 level.

Table S5. Comparison of effects of different oat varieties on soil Mg^{2+}

variety	0-15cm soil Mg^{2+}/(g·kg^{-1})		15-30cm soil Mg^{2+}/（g·kg^{-1}）	
	**Seedling stage**	**Harvest period**	**Seedling stage**	**Harvest period**
CK	0.09±0.01a	0.10±0.00a	0.10±0.01a	0.08±0.00a
Qingtian No. 1	0.03±0.01gh	0.01±0.00e	0.03±0.01efg	0.01±0.01f
Molasses	0.02±0.00gh	0.01±0.00e	0.02±0.00g	0.01±0.01ef
Qinghai sweet oat	0.04±0.01efg	0.03±0.01bcd	0.05±0.01de	0.02±0.01ef
Qingyin No. 1	0.03±0.00fgh	0.03±0.01cde	0.02±0.01fg	0.01±0.00ef
Qingyin No. 2	0.08±0.01ab	0.02±0.00de	0.04±0.00def	0.02±0.01def
Dafuweng	0.05±0.01cde	0.02±0.01de	0.07±0.01c	0.03±0.01cdef
Baiyan No. 7	0.07±0.01bc	0.04±0.00b	0.08±0.01bc	0.07±0.01a
Northwestern No. 1	0.06±0.01bcd	0.04±0.01bc	0.05±0.01d	0.05±0.01b
Lena	0.05±0.01def	0.04±0.01bc	0.04±0.01def	0.04±0.00bcd
Jiayan No. 2	0.02±0.00h	0.05±0.01b	0.04±0.01def	0.04±0.01bc
Mengshi No. 1	0.05±0.02cde	0.02±0.01de	0.04±0.01def	0.02±0.00ef
Qinghai No. 444	0.06±0.01bcd	0.01±0.00e	0.02±0.01g	0.02±0.01def
Haymaker	0.07±0.00b	0.02±0.00e	0.04±0.00de	0.02±0.01def
Gaoyan No. 1	0.04±0.00efg	0.02±0.01cde	0.09±0.01ab	0.03±0.01bcde
Baler	0.03±0.01fgh	0.02±0.01de	0.03±0.00defg	0.03±0.01bcd
F Value	12.00**	14.77**	15.76**	11.39**

Note: CK is saline-alkali open space control. The values in the table are mean±standard error, different letters indicate significant differences among different breeds in the same column (P<0.05). ** indicates highly significant differences at the 0.01 level; * indicates significant differences at the 0.05 level.

Table S6. Comparison of effects of different oat varieties on soil HCO_3^-

variety	0-15cm soil HCO_3^-/(g·kg^{-1})		15-30cm soil HCO_3^-/(g·kg^{-1})	
	**Seedling stage**	**Harvest period**	**Seedling stage**	**Harvest period**
CK	0.31±0.01a	0.22±0.01a	0.26±0.01a	0.18±0.00cd
Qingtian No. 1	0.28±0.01b	0.18±0.00efg	0.21±0.01ef	0.17±0.01d
Molasses	0.25±0.01cde	0.19±0.01cdef	0.25±0.00ab	0.23±0.00a
Qinghai sweet oat	0.24±0.00de	0.17±0.01fg	0.22±0.01cde	0.17±0.00d
Qingyin No. 1	0.23±0.01efg	0.17±0.00g	0.22±0.01cde	0.21±0.01b
Qingyin No. 2	0.24±0.01ef	0.20±0.01bcde	0.22±0.00cde	0.17±0.00cd
Dafuweng	0.20±0.01i	0.19±0.01defg	0.22±0.00def	0.18±0.01cd
Baiyan No. 7	0.26±0.01cd	0.21±0.01ab	0.22±0.00cde	0.15±0.00e
Northwestern No. 1	0.24±0.01de	0.19±0.01defg	0.21±0.01ef	0.18±0.00cd
Lena	0.27±0.00bc	0.18±0.01efg	0.21±0.01def	0.17±0.01cd
Jiayan No. 2	0.22±0.00gh	0.18±0.01fg	0.23±0.00cd	0.17±0.01d
Mengshi No. 1	0.20±0.01hi	0.19±0.01defg	0.24±0.01bc	0.21±0.01b
Qinghai No. 444	0.27±0.01bc	0.20±0.01bcde	0.21±0.01ef	0.19±0.00c
Haymaker	0.22±0.01fgh	0.19±0.01cdef	0.21±0.01def	0.21±0.01b
Gaoyan No. 1	0.23±0.01efg	0.21±0.00abc	0.20±0.01f	0.17±0.01d
Baler	0.23±0.01efg	0.21±0.00abcd	0.21±0.01ef	0.18±0.01cd
F Value	20.86**	4.72**	7.11**	18.07**

Note: CK is saline-alkali open space control. The values in the table are mean±standard error, different letters indicate significant differences among different breeds in the same column (P<0.05). ** indicates highly significant differences at the 0.01 level; * indicates significant differences at the 0.05 level.

Table S7. Comparison of the effects of different oat varieties on soil K^+

variety	0-15cm soil K^+/(g·kg^{-1})		15-30cm soil K^+/(g·kg^{-1})	
	**Seedling stage**	**Harvest period**	**Seedling stage**	**Harvest period**
CK	0.19±0.02a	0.17±0.02a	0.09±0.01a	0.08±0.01a
Qingtian No. 1	0.10±0.02ef	0.03±0.02h	0.10±0.01a	0.06±0.01bcd
Molasses	0.11±0.01de	0.04±0.02gh	0.06±0.01bcd	0.05±0.02bcd
Qinghai sweet oat	0.13±0.02bcd	0.13±0.01bc	0.06±0.01bcd	0.03±0.02efg
Qingyin No. 1	0.16±0.00b	0.06±0.00fgh	0.06±0.00bc	0.05±0.02bcde
Qingyin No. 2	0.05±0.01gh	0.08±0.02ef	0.02±0.00fg	0.03±0.01defg
Dafuweng	0.12±0.02cde	0.03±0.02h	0.05±0.02cd	0.02±0.01g
Baiyan No. 7	0.10±0.01ef	0.07±0.02efg	0.07±0.00b	0.02±0.00fg
Northwestern No. 1	0.07±0.02fg	0.16±0.00ab	0.03±0.00ef	0.07±0.01ab
Lena	0.08±0.00fg	0.12±0.01bcd	0.03±0.01ef	0.04±0.01defg
Jiayan No. 2	0.05±0.01gh	0.13±0.00b	0.02±0.00fg	0.04±0.01cdef
Mengshi No. 1	0.11±0.01de	0.14±0.02ab	0.05±0.01bcd	0.07±0.01abc
Qinghai No. 444	0.06±0.01gh	0.08±0.02ef	0.02±0.01fg	0.03±0.01efg
Haymaker	0.03±0.01h	0.10±0.00cde	0.01±0.00g	0.02±0.00fg
Gaoyan No. 1	0.14±0.02bc	0.09±0.02def	0.05±0.00cd	0.05±0.01bcde
Baler	0.06±0.00gh	0.04±0.02gh	0.04±0.01de	0.02±0.01fg
F Value	17.08**	14.51**	19.83**	5.70**

Note: CK is saline-alkali open space control. The values in the table are mean±standard error, different letters indicate significant differences among different breeds in the same column ($P<0.05$). ** indicates highly significant differences at the 0.01 level; * indicates significant differences at the 0.05 level.

Table S8. Comparison of effects of different oat varieties on soil Na^+

variety	0-15cm soil $Na^+/(g\cdot kg^{-1})$		15-30cm soil $Na^+/(g\cdot kg^{-1})$	
	**Seedling stage**	**Harvest period**	**Seedling stage**	**Harvest period**
CK	0.54±0.01a	0.41±0.02a	0.32±0.02a	0.31±0.01a
Qingtian No. 1	0.03±0.02i	0.15±0.02def	0.04±0.02h	0.16±0.02e
Molasses	0.06±0.01gh	0.06±0.01h	0.10±0.00ef	0.03±0.01h
Qinghai sweet oat	0.18±0.02d	0.15±0.00def	0.16±0.02cd	0.06±0.00h
Qingyin No. 1	0.13±0.00e	0.13±0.02f	0.09±0.02fg	0.04±0.02h
Qingyin No. 2	0.22±0.01c	0.08±0.02gh	0.13±0.01de	0.10±0.02g
Dafuweng	0.20±0.01cd	0.07±0.01gh	0.22±0.02b	0.09±0.01g
Baiyan No. 7	0.34±0.00b	0.17±0.02de	0.18±0.01c	0.17±0.02e
Northwestern No. 1	0.19±0.01cd	0.18±0.02d	0.29±0.02a	0.27±0.01bc
Lena	0.19±0.01cd	0.21±0.01c	0.12±0.02de	0.14±0.02ef
Jiayan No. 2	0.08±0.01fg	0.18±0.01de	0.15±0.01ef	0.24±0.01c
Mengshi No. 1	0.07±0.02fgh	0.22±0.01c	0.11±0.01ef	0.17±0.02de
Qinghai No. 444	0.05±0.01hi	0.23±0.02c	0.09±0.01ef	0.10±0.01g
Haymaker	0.03±0.00i	0.27±0.02b	0.06±0.01gh	0.20±0.01d
Gaoyan No. 1	0.10±0.00ef	0.15±0.01ef	0.13±0.02de	0.29±0.00ab
Baler	0.10±0.00f	0.09±0.01g	0.16±0.02cd	0.12±0.00fg
F Value	202.94**	62.38**	39.45**	79.54**

Note: CK is saline-alkali open space control. The values in the table are mean±standard error, different letters indicate significant differences among different breeds in the same column (P<0.05). ** indicates highly significant differences at the 0.01 level; * indicates significant differences at the 0.05 level.

Table S9. Comparison of effects of different oat varieties on soil Cl^-

variety	0-15cm soil $Cl^-/(g·kg^{-1})$		15-30cm soil $Cl^-/(g·kg^{-1})$	
	**Seedling stage**	**Harvest period**	**Seedling stage**	**Harvest period**
CK	0.44±0.01a	0.50±0.01a	0.56±0.01a	0.66±0.01a
Qingtian No. 1	0.06±0.01i	0.23±0.01gh	0.06±0.01m	0.26±0.01fg
Molasses	0.09±0.00hi	0.12±0.01i	0.06±0.00m	0.11±0.01k
Qinghai sweet oat	0.34±0.01c	0.32±0.01def	0.35±0.01d	0.11±0.01k
Qingyin No. 1	0.24±0.01f	0.28±0.01efg	0.07±0.01m	0.17±0.00i
Qingyin No. 2	0.09±0.01hi	0.50±0.01a	0.23±0.00h	0.23±0.01hi
Dafuweng	0.29±0.01d	0.13±0.01i	0.38±0.01c	0.22±0.01i
Baiyan No. 7	0.36±0.01b	0.35±0.00cd	0.49±0.00d	0.34±0.01e
Northwestern No. 1	0.29±0.01d	0.34±0.01cdef	0.32±0.00e	0.39±0.01d
Lena	0.27±0.01e	0.38±0.00bcd	0.25±0.01g	0.33±0.01e
Jiayan No. 2	0.09±0.00hi	0.43±0.00ab	0.30±0.00f	0.59±0.01b
Mengshi No. 1	0.08±0.00hi	0.40±0.15bc	0.15±0.01k	0.27±0.01f
Qinghai No. 444	0.08±0.01i	0.35±0.01cde	0.09±0.01l	0.16±0.01i
Haymaker	0.04±0.00j	0.40±0.00bc	0.04±0.00n	0.25±0.01gh
Gaoyan No. 1	0.12±0.01g	0.26±0.00fg	0.17±0.00j	0.56±0.01c
Baler	0.10±0.00h	0.18±0.01hi	0.20±0.00i	0.22±0.01i
F Value	416.07^{**}	19.57^{**}	1255.09^{**}	676.68^{**}

Note: CK is saline-alkali open space control. The values in the table are mean±standard error, different letters indicate significant differences among different breeds in the same column ($P<0.05$). ** indicates highly significant differences at the 0.01 level; * indicates significant differences at the 0.05 level.

Table S10. Comparison of the effects of different oat varieties on soil SO_4^{2-}

variety	0-15cm soil SO_4^{2-}/(g·kg^{-1})		15-30cm soil SO_4^{2-}/(g·kg^{-1})	
	Seedling stage	Harvest period	Seedling stage	Harvest period
CK	0.54±0.01a	0.65±0.02a	0.52±0.02a	0.57±0.02a
Qingtian No. 1	0.24±0.01ef	0.19±0.01f	0.24±0.03ef	0.19±0.01f
Molasses	0.13±0.02h	0.03±0.01g	0.15±0.01ij	0.00±0.00i
Qinghai sweet oat	0.22±0.01f	0.21±0.01f	0.32±0.02d	0.01±0.02hi
Qingyin No. 1	0.22±0.00f	0.21±0.02f	0.12±0.01i	0.04±0.02h
Qingyin No. 2	0.08±0.02i	0.51±0.00bc	0.16±0.01i	0.19±0.01f
Dafuweng	0.36±0.02c	0.18±0.01f	0.42±0.01c	0.14±0.02g
Baiyan No. 7	0.42±0.01b	0.35±0.02e	0.48±0.02b	0.29±0.01e
Northwestern No. 1	0.34±0.01c	0.46±0.03d	0.31±0.00d	0.50±0.01b
Lena	0.29±0.01d	0.32±0.01e	0.20±0.01gh	0.32±0.02de
Jiayan No. 2	0.09±0.01i	0.53±0.02b	0.18±0.02hi	0.39±0.02c
Mengshi No. 1	0.09±0.01i	0.43±0.02d	0.17±0.01hi	0.34±0.02d
Qinghai No. 444	0.09±0.02i	0.52±0.01bc	0.12±0.02i	0.21±0.01f
Haymaker	0.08±0.02i	0.50±0.02c	0.21±0.01fg	0.42±0.02c
Gaoyan No. 1	0.26±0.01e	0.43±0.02d	0.20±0.01gh	0.55±0.02a
Baler	0.19±0.01e	0.33±0.02e	0.26±0.01e	0.35±0.02f
F Value	193.02**	190.24**	122.29**	228.70**

Note: CK is saline-alkali open space control. The values in the table are mean±standard error, different letters indicate significant differences among different breeds in the same column (P<0.05). ** indicates highly significant differences at the 0.01 level; * indicates significant differences at the 0.05 level.

Table S11. Subordinate function values of soil physical and chemical properties of different oat varieties at harvest period

Variety	soil pH	soil bulk electrical conductivity	Soil organic matter	Soil total nitrogen	soil total salt	Ca^{2+}	Na^{+}	K^{+}	Mg^{2+}	Cl^{-}	HCO_3^{-}	SO_4^{2-}	Mean
A	0.116	0.944	0.935	0.788	0.646	1.000	0.419	0.795	1.000	0.674	0.904	0.639	0.738
B	0.254	1.000	0.481	0.349	0.834	0.994	1.000	0.750	0.960	1.000	0.000	1.000	0.719
C	0.713	0.556	0.977	0.616	0.762	0.468	0.684	0.406	0.734	0.750	0.978	0.796	0.703
D	0.616	0.556	0.988	0.815	0.826	0.657	0.777	0.669	0.818	0.737	0.587	0.769	0.735
E	0.649	0.667	0.714	0.315	0.591	0.649	0.759	0.637	0.825	0.367	0.609	0.288	0.589
F	0.806	0.722	0.276	0.240	0.760	0.502	0.809	1.000	0.713	0.863	0.709	0.692	0.674
G	0.789	0.778	0.609	0.897	0.149	0.000	0.351	0.778	0.000	0.424	0.687	0.359	0.485
H	1.000	0.000	1.000	0.596	0.000	0.058	0.050	0.000	0.322	0.382	0.687	0.031	0.344
I	0.886	0.389	0.141	0.000	0.409	0.459	0.292	0.369	0.453	0.397	0.787	0.352	0.411
J	0.655	0.667	0.437	0.363	0.759	0.427	0.124	0.292	0.268	0.000	1.000	0.064	0.421
K	0.060	0.667	0.752	0.322	0.750	0.808	0.204	0.143	0.891	0.451	0.261	0.225	0.461
L	0.000	0.833	0.000	0.226	0.993	0.746	0.344	0.700	0.836	0.652	0.413	0.264	0.501
M	0.093	0.833	0.201	0.459	1.000	0.504	0.000	0.616	0.883	0.478	0.204	0.071	0.445
N	0.079	0.333	0.873	1.000	0.625	0.093	0.071	0.495	0.673	0.255	0.470	0.000	0.414
O	0.114	0.278	0.800	0.425	0.582	0.457	0.681	0.950	0.652	0.802	0.422	0.321	0.540

Note : soil organic matter, soil total nitrogen index are positive correlation index, soil pH, soil bulk electrical conductivity, soil total salt, Ca^{2+}、Na^{+}、K^{+}、Mg^{2+}、Cl^{-}、HCO_3^{-}、SO_4^{2-} are negative correlation index. A to O are respectively Qingtian No. 1, Molasses, Qinghai sweet oat, Qingyin No. 1, Qingyin No. 2, Dafuweng, Baiyan No. 7, Northwestern No. 1, Lena, Jiayan No. 2, Mengshi No. 1, Qinghai No. 444, Haymaker, Gaoyan No. 1, Baler.
